# EMDB—the Electron Microscopy Data Bank

**DOI:** 10.1093/nar/gkad1019

**Published:** 2023-11-22

**Authors:** The wwPDB Consortium, Jack Turner, Sanja Abbott, Neli Fonseca, Ryan Pye, Lucas Carrijo, Amudha Kumari Duraisamy, Osman Salih, Zhe Wang, Gerard J Kleywegt, Kyle L Morris, Ardan Patwardhan, Stephen K Burley, Gregg Crichlow, Zukang Feng, Justin W Flatt, Sutapa Ghosh, Brian P Hudson, Catherine L Lawson, Yuhe Liang, Ezra Peisach, Irina Persikova, Monica Sekharan, Chenghua Shao, Jasmine Young, Sameer Velankar, David Armstrong, Marcus Bage, Wesley Morellato Bueno, Genevieve Evans, Romana Gaborova, Sudakshina Ganguly, Deepti Gupta, Deborah Harrus, Ahsan Tanweer, Manju Bansal, Vetriselvi Rangannan, Genji Kurisu, Hasumi Cho, Yasuyo Ikegawa, Yumiko Kengaku, Ju Yaen Kim, Satomi Niwa, Junko Sato, Ayako Takuwa, Jian Yu, Jeffrey C Hoch, Kumaran Baskaran, Wenqing Xu, Weizhe Zhang, Xiaodan Ma

## Abstract

The Electron Microscopy Data Bank (EMDB) is the global public archive of three-dimensional electron microscopy (3DEM) maps of biological specimens derived from transmission electron microscopy experiments. As of 2021, EMDB is managed by the Worldwide Protein Data Bank consortium (wwPDB; wwpdb.org) as a wwPDB Core Archive, and the EMDB team is a core member of the consortium. Today, EMDB houses over 30 000 entries with maps containing macromolecules, complexes, viruses, organelles and cells. Herein, we provide an overview of the rapidly growing EMDB archive, including its current holdings, recent updates, and future plans.

## Graphical Abstract

**Graphical Abstract:**
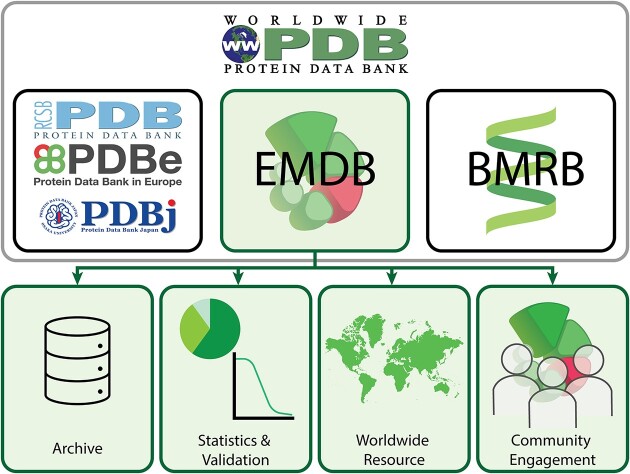

## Introduction

In the past decade, 3DEM techniques including cryogenic-sample electron microscopy and tomography (cryoEM and cryoET, respectively) (1), have become leading structure-determination techniques in the field of structural biology (2–4). Since the advent of the ‘Resolution Revolution’ (5) 3DEM is routinely being used to study biological structures on scales from atoms (6,7) to molecules in cells (8,9), to whole cells (e.g. EMD-11073 (10)). In addition, 3DEM has proved valuable for the study of sample heterogeneity, as is well reviewed in (11), and new software is pushing the study of protein heterogeneity even further (12–16).

In 2002, the Macromolecular Structure Database group (from which resources such as Electron Microscopy Data Bank (EMDB) (17), Electron Microscopy Public Image Archive (EMPIAR) (18) and the Protein Data Bank in Europe (PDBe) (19) have emerged) established the EMDB for the archiving and dissemination of 3DEM volumes (20). EMDB is located at the European Molecular Biology Laboratory's European Bioinformatics Institute (EMBL-EBI) in Hinxton, UK. In 2007, the Research Collaboratory for Structural Bioinformatics (RCSB) (21) and the National Center for Macromolecular Imaging (NCMI) (22) joined forces with the EMDB. The Protein Data Bank Japan (PDBj) (23) joined this collaboration in 2013. In 2021, the EMDB archive became a core wwPDB archive alongside the PDB (24) and BMRB (25), with the EMDB team at EMBL-EBI becoming a core wwPDB member and serving as the wwPDB-designated Archive Keeper for the EMDB core archive. Protein Data Bank China (PDBc) recently joined the wwPDB as an associate member (26), further expanding the international collaboration managing these archives. wwPDB core archives are made available at no charge and with no limitation on usage under the CC0 1.0 Creative Commons licence (https://creativecommons.org/share-your-work/public-domain/cc0/). All three core archives managed jointly by the wwPDB operate under the FAIR principles of Findability, Accessibility, Interoperability and Reusability (27). Following the FAIR principles facilitates data discovery, collaboration, and data reproducibility—all of which are important for accelerating research and innovation.

Interoperation between archives is essential for serving the complete data associated with a 3DEM experiment. The 3DEM Coulomb potential map (henceforth referred to as ‘map’ or ‘volume’) is stored in the EMDB archive, whereas derived atomic coordinate structures are stored in the PDB archive. The raw data from which maps are derived are collected by the Electron Microscopy Public Image Archive (EMPIAR) (18). In addition to the aforementioned structural archives, entries in EMDB can also be associated with entries in the AlphaFold Protein Structure Database (28), Small Angle Scattering Biological Data Bank (SASBDB) (29), PDB-Dev (30) and EMPIAR (18).

The wwPDB members support its core mission of sustaining freely accessible, interoperating Core Archives of structure data and metadata for biological macromolecules as an enduring public good to promote basic and applied research and education across the sciences (24). Here, we provide an overview of the recent developments in the EMDB archive including its content, deposition protocols, use and future prospects.

## Archive content

### Growth and statistics

EMDB archives volumes from single-particle analysis (SPA), subtomogram averaging (STA), helical reconstruction (HR), tomography, and electron crystallography (EC). All these techniques produce 3D volume data with the exception of EC, which produces diffraction data from which such volumes can be calculated.

**Figure 1.**
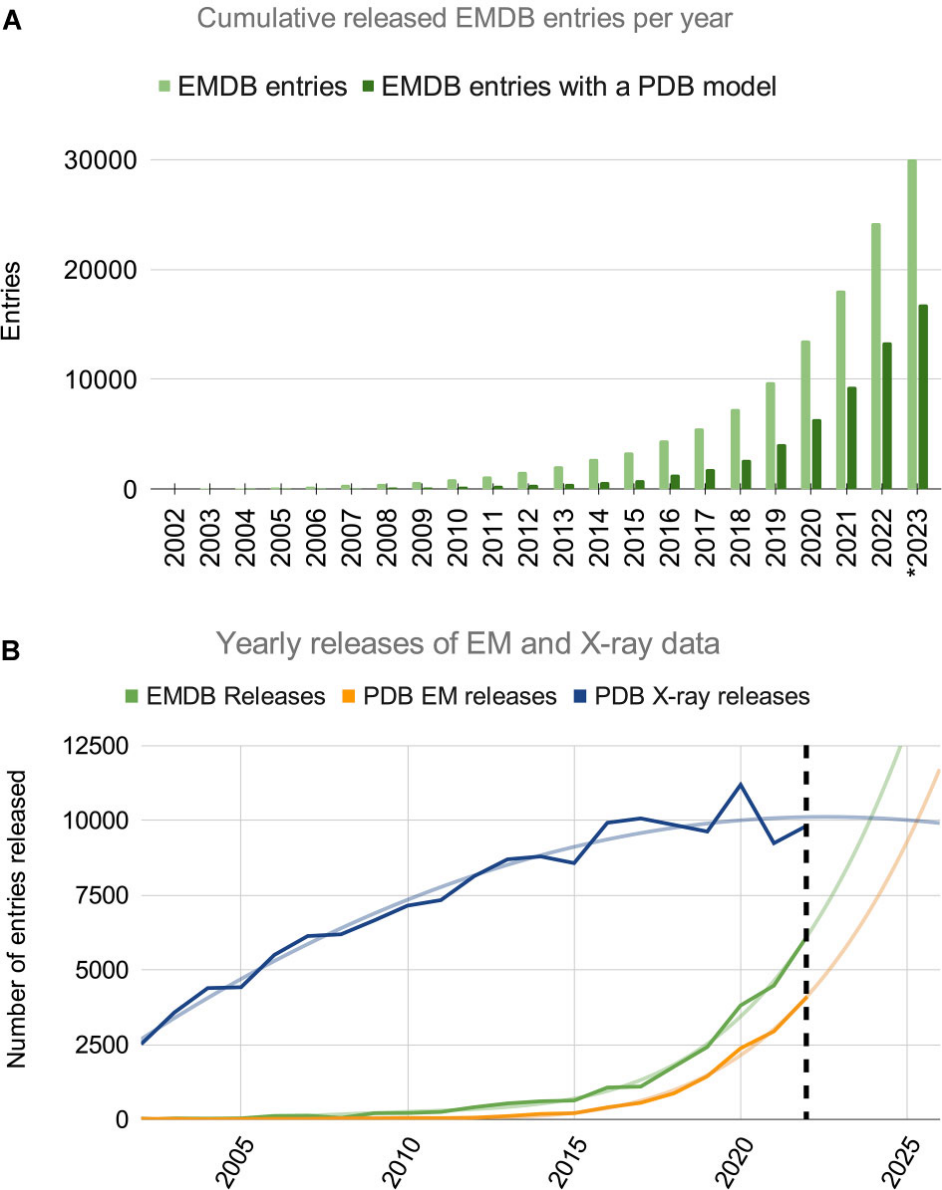
(**A**) Cumulative number of all EMDB entries and of those with an atomic coordinate structure in the PDB at the end of each year. *2023 data are through October 4th. (**B**) Number of annually released EMDB and PDB entries. EMDB data includes all modalities. PDB data is split according to X-ray and EM models released per year. Data is shown until the end of 2022 (dashed black line) with trendlines showing predicted future growth.

EMDB holds >30 000 entries as of 4 October 2023, approximately 55% of which have associated atomic coordinates archived in the PDB (Figure 1A). The number of 3DEM entries released per year is growing exponentially (Figure 1b). If this growth continues EMDB is predicted to hold 50 000 entries in 2025 and 100 000 entries in 2028 (at the current archive-doubling time of ∼2.5 years), with ∼13 500 and ∼31 500 releases predicted in these years, respectively. Based on the current trends it is expected that the number of PDB 3DEM entries released per year will surpass PDB Macromolecular Crystallography or MX releases in 2025. The rapid growth of 3DEM entries highlights the increased accessibility and utility of 3DEM methods.

**Figure 2.**
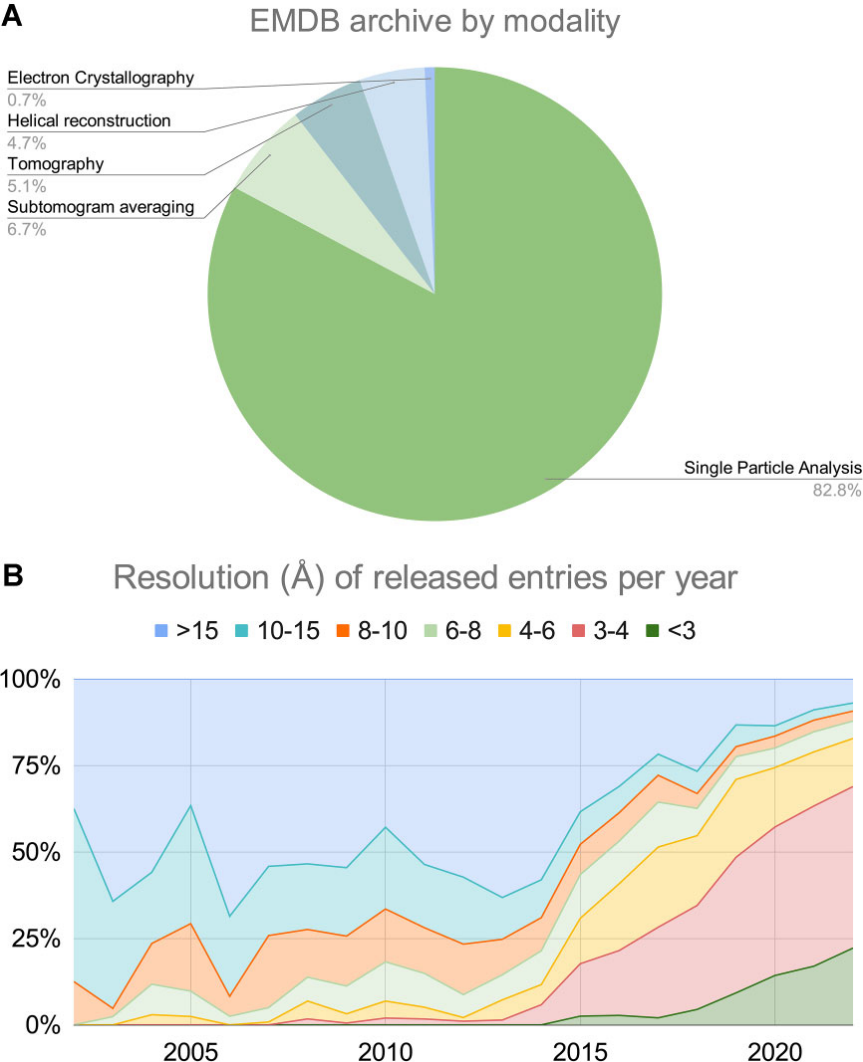
(**A**) Proportion of the EMDB archive contributed by each modality as of the end of 2022. (**B**) Proportion of released entries in various resolution (Å) shells per year from 2002 to 2022. Note the huge impact of the resolution revolution since 2014.

The fraction of entries in the EMDB archive determined by each modality is shown in Figure 2A. SPA is by far the most popular method used to generate volumes that are deposited to the archive, making up 82.8% of the total archive at the end of 2022, an increase of >4% since 2016 (17). STA, Tomography, HR, and EC make up the remaining 17.2% in descending order. Using metadata harvested from EMDB, the effect of the resolution revolution (5) can be visualised (Figure 2B). The biological insight being sought by the researcher will ultimately dictate the resolution that is required from the 3DEM volume obtained from the investigation. Macromolecular structure, interactions and function may be sufficiently described by ‘lower resolution’ volumes. In 2022 alone, 307 SPA maps equal to or below 10 Å resolution were released, each providing novel insights to the scientific community. Where the goal of the investigation is to model a macromolecular structure with atomic accuracy, a high resolution 3DEM reconstruction is sought. More than 60% of entries in 2022 were at better than 4 Å resolution (4034 out of 6139) and over 20% of entries from the same year were sub-3 Å (1299 out of 6139). At present, the archive also contains structures with atomic resolvability (<1.2 Å, as per the Sheldrick criterion (31,32)), including 2 SPA and 28 EC entries.

### Archive data

Each EMDB entry describes a macromolecular complex or subcellular structure represented in a 3DEM volume. In certain cases, multiple entries may be linked to fully represent the potential multiple volumes output from a 3DEM experiment. All entries contain a primary 3DEM volume around which an entry is based. Unfiltered, unmasked and unsharpened half-maps, generated by default in most SPA, STA and HR workflows, have been mandatory for all relevant modalities deposited since 25 February 2022 (https://www.wwpdb.org/news/news?year=2022#6218da3152988f064bf8c4a3). Each entry contains an image provided by the depositor, enabling them to showcase the primary volume according to their preferences. A number of optional additional files may also be present in an entry, including additional volumes, masks, a Fourier Shell Correlation (FSC) curve, and layer-line files. Table 1 gives an overview of all file types that may be present in an entry.

**Table 1.** An overview of possible entry content held in the EMDB archive

Category (number)	Description
Primary map (1)	EM map or tomogram that is described in the associated publication
Half-maps (0 or 2)^a^	Unfiltered, unsharpened, and unmasked raw half-maps for SPA, SPA based helical reconstructions, or STA
Masks (0 or more)	Primary/raw map masks, segmentation/focused-refinement masks, and half-map masks
Additional cryo-EM maps (0 or more)	Examples include difference maps, maps showing alternative conformational states and/or compositions, and maps with different processing (for example filtering, sharpening, and masking)
Auxiliary files (0 or more)	Examples include author-determined FSC curves (half-maps, map–model, …), structure factors, and layer lines
Metadata files (2)	XML and mmCIF files containing an entry's metadata
Validation Report (1)	Volume-only validation reports (3 file formats).

^a^Deposition of half-maps was made mandatory in February of 2022.

An entry's primary volume must be adequately described with additional metadata to ensure adherence to FAIR principles (27). These metadata are stored in Extensible Markup Language (XML) and mmCIF files with items and attributes defined in the EMDB data model (www.ebi.ac.uk/emdb/documentation#version30) and mmCIF dictionary (mmcif.wwpdb.org), respectively. These definitions include a hierarchy of information which allows description of cellular structures, supramolecules, and macromolecules, all of which may exist in a single volume. Various other types of experimental metadata are also present in these files, including information on specimen preparation, microscopy instrumentation, data-collection protocol, and software used during volume generation and processing. The EMDB data model includes definitions of mandatory items and support for enumerations and allowed data ranges. All metadata files in the EMDB archive can, therefore, be validated against the XML schema.

## Global data deposition

Depositions to EMDB are managed by the wwPDB global OneDep deposition (33), validation (34–36), and biocuration software system (37). OneDep is a unified deposition system for 3DEM, X-ray, and NMR 3D biostructures, experimental data and related metadata. OneDep is hosted at wwPDB data centres around the world (located in the USA, Europe and Asia) providing a consistent deposition experience independent of geographic location. Biocuration of incoming entries is geographically distributed among wwPDB partner sites as follows: RCSB PDB handles all depositions from the Americas and Oceania, PDBe and the EMDB team at EMBL-EBI manage depositions from Europe and Africa, and PDBj and PDBc handle all depositions from Asia. This arrangement divides the effort of biocuration and ensures that depositors can communicate with wwPDB biocurators within, or close to, their local timezone.

The OneDep system makes use of the PDBx/mmCIF framework during deposition, validation, and biocuration. All data and metadata related to a deposition are defined in the EMDB data model, which in turn informs the PDBx/mmCIF dictionary (mmcif.wwpdb.org) (38). Thus for 3DEM, the PDBx/mmCIF dictionary faithfully represents the EMDB model described in the EMDB XML schema. Data specific to 3DEM methods in the PDBx/mmCIF dictionary use the ‘em’ namespace (*e.g*. ‘em_imaging’ for metadata on the electron microscope setup (mmcif.wwpdb.org/dictionaries/mmcif_pdbx_v50.dic/ Categories/em_imaging.html)). The dictionary definitions can also include rules such as relationships between different data items, enumerations, and allowed ranges. Finally, the PDBx/mmCIF format is extensible, allowing the dictionary to grow with the data models for all three wwPDB core archives (PDB, EMDB and BMRB).

At the end of the deposition, validation and biocuration processes, a wwPDB validation report for reviewers is provided to the depositor. This report contains a range of community-recommended validation metrics for both the volume and, if present, the atomic coordinates (34,36,39). It is strongly recommended by wwPDB that depositors provide their confidential post-biocuration wwPDB validation reports in PDF format to scientific journals when submitting related manuscripts (24). Validation metrics related to 3DEM volumes are implemented in a tiered approach (36), allowing testing and gathering of community feedback by presenting the metrics on the EMDB website (tiers 1 and 2) before potential implementation in the wwPDB validation reports (tier 3). *Q*-score (40) is the most recently added tier-3 metric, providing a new metric which complements the previously implemented atom-inclusion score (41).

Upon depositor request, manuscript publication (including on a preprint server), or when one year has elapsed since the date of deposition, an entry is prepared for public release in the next weekly EMDB update cycle. Entries can be prepared for release from Monday to Thursday in any given week. Release of atomic coordinates cannot precede release of the associated EMDB entry. Every Thursday, after all entries are ready for release, the EMDB archive begins the release process, which includes data-integrity checks, running of automated validation and data-enrichment pipelines, and manual inspection of validation outputs for all entries. Depositors are contacted in the event of any peculiarities noticed during final validation checks. New data are then made public every Wednesday at 00:00 Universal Time Coordinated or UTC.

## Data dissemination

**Figure 3.**
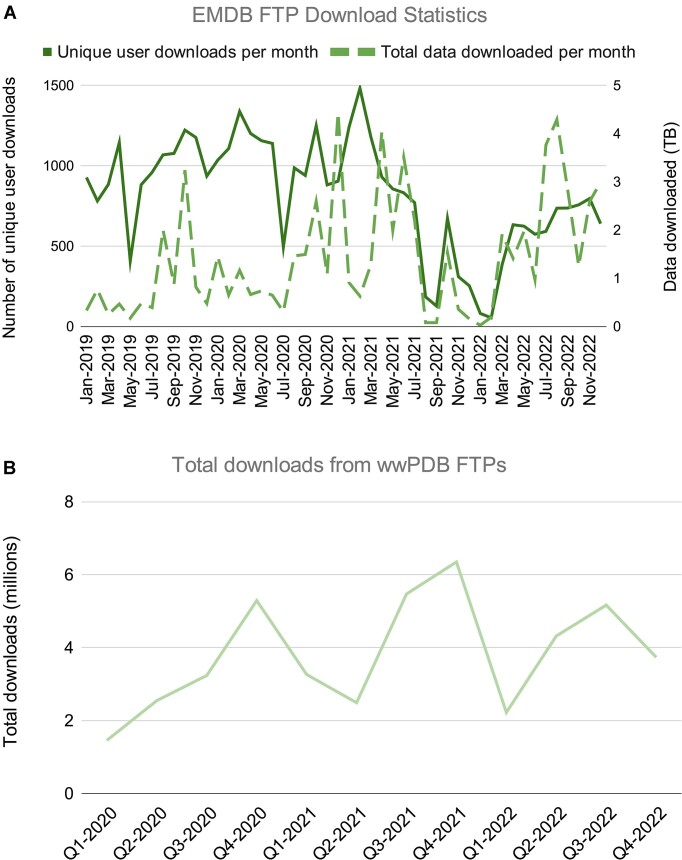
(**A**) Monthly counts of the number of downloads by unique users each month (solid line, left axis) and the total amount of data served (dashed line, right axis). (**B**) Quarterly counts of the total number of downloads of EMDB data from all wwPDB FTP sites.

The EMDB archive is served *via* an FTP site (https://ftp.ebi.ac.uk/pub/databases/emdb/), which is mirrored by wwPDB (https://files.wwpdb.org/pub/emdb), RCSB PDB (https://files.rcsb.org/pub/emdb) and PDBj (https://files.pdbj.org/pub/emdb/). The EMDB FTP site provides access to volumes, entry images, FSC data, metadata, and validation files for released entries. The EMDB FTP site also serves files describing the EMDB data model and the status of all released and unreleased 3DEM entries in the ‘/doc/’ and ‘/status/’ folders of the EMDB FTP area, respectively. The number of unique IP addresses from which downloads are initiated in a given calendar month and total data downloaded are shown in Figure 3A. In 2022, total volume of downloaded data exceeded 24 TB (the total archive size at the end of 2022 was 8.9 TB), a 33% increase on the previous year. When assessing total downloads of EMDB data across all wwPDB FTP sites (Figure 3B) a seasonal variation is observed. In 2020 and 2021, most downloads occurred during Q4, whereas in 2022 download requests peaked slightly earlier, in Q3.

Data, news, and statistics relevant to EMDB can be found on the EMDB website (ebi.ac.uk/emdb/). News and statistics pertaining to the wider wwPDB, including EMDB, are available on the wwPDB website (wwpdb.org). Volume data is served in CCP4/MRC map format (ftp.ebi.ac.uk/pub/databases/emdb/doc/Map-format/). Metadata is provided in XML and mmCIF format, with the EMDB data model described in the ‘docs/’ section of the EMDB FTP site.

## Archive updates

A major change to the EMDB archive involved the transition to a new version of the EMDB data model, which underpins the data structure of the archive. The new data model, fully implemented in 2021, contains a rich set of metadata and supports hierarchical description of the sample composition. Descriptions of both molecular and cellular samples are supported and metadata has been modularised such that different 3DEM modalities have specific data items. Taken together these features facilitate a more accurate and complete description of 3DEM entries in the EMDB archive relative to the previous version of the data model. The schema and documentation for the data model are available online (https://www.ebi.ac.uk/emdb/documentation).

When extending the EMDB data model, archive remediation is required to add new information to legacy entries, where possible. One recent addition to the data model is the option to link EMDB entries to SASBDB entries. These data are now available for newly released entries and a remediation will be carried out to add this information for relevant legacy entries. When major changes are made to the archive or policies, they are communicated via the EMDB website and wwPDB channels, including emails to major mailing lists (e.g. CCPEM and 3DEM).

## Community engagement

Community engagement is at the heart of archive management, such as annotation practices, archive policies and future development plans. To this end, wwPDB is guided by an expert international advisory committee. The committee advises on the activities of the wwPDB partners and outcomes of its annual meetings are published on the wwPDB website (http://www.wwpdb.org/about/advisory).

In addition to the annual advisory committee meetings, several workshops specific to the planning of EMDB’s future endeavours have been conducted. Community experts were engaged for the purposes of ensuring optimal data management (42), annotation of cellular data (43) and ensuring integration of data between multiple resources (44). The validation methods in 3DEM are constantly evolving, and no one metric can provide an overall description of volume quality. An expert Validation Task Force was set up and first met in 2010 (39), with a second meeting held in 2020. This group contributes and advises on the validation metrics EMDB includes in its Validation Analysis resource (36) and the wwPDB validation reports (33).

The EMDB team is committed to training new scientists in EMDB and OneDep, and communicating science via a number of outreach channels. EMDB staff members have given lectures at various training courses around the world. They also run an X (previously Twitter) and YouTube channel to highlight recently released entries and share EMDB news.

## Current trends and future outlook

### Rapid growth

**Figure 4.**
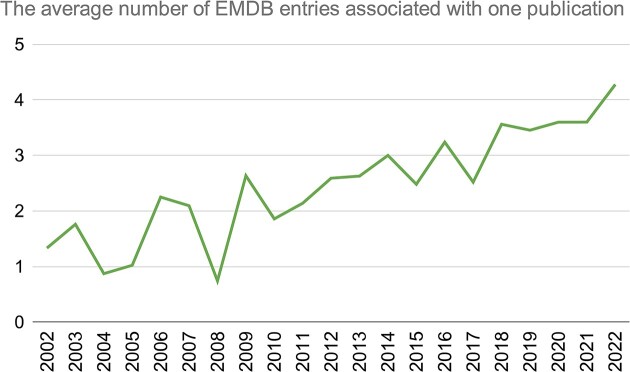
The average number of EMDB entries associated with a single publication per year.

The EMDB archive continues to experience exponential growth year-on-year (Figure 1), while at the same time depositors are creating more archive entries per publication (Figure 4). The wwPDB partners are addressing these developments through improvements to the OneDep deposition, validation and biocuration system. Biocuration and validation workflows are under continuous review, to inform, plan, design and implement improvements such as increased automation. Depositor experience and efficiency are also important considerations. The ORCID-based login feature allows depositors to access all their depositions within their browser, rather than having to keep a logbook of all deposition IDs. A deposition API is also in development, which will allow software packages to directly deposit data to the OneDep system. In the most favourable cases, this could entirely obviate the need for depositor intervention, bringing a level of efficiency and convenience to the deposition process that is necessary to accommodate the growth of 3DEM volume generation by the community. Programmatic collection and deposition of data also represents a method for improving metadata accuracy and completeness. wwPDB is currently working with developers from popular structural biology software packages to accelerate the adoption of the deposition API.

### Rapidly evolving science

Every 3DEM modality supported by EMDB is experiencing continuous and rapid innovation and improvement. Single particle-based methods (SPA and STA) can now be used to analyse continuous conformational variability of a macromolecule, sometimes referred to as heterogeneity analysis (12–15). These approaches allow many structural states to be resolved using a single imaging dataset. Simultaneously, tomography is seeing explosive growth in the amount of data that can be recorded per unit time (45–47). Such advances require frequent updates to the EMDB data model and PDBx/mmCIF dictionary. wwPDB plans to establish a wwPDB 3DEM working group to accelerate implementation of such additions and promote adoption of data and methodological standards across the community.

Software development in the field of artificial intelligence and machine learning (AI/ML) has been nothing short of spectacular in recent years. EMDB data have already been used in the development of new AI-based software tools for various applications, including particle picking (48), particle pruning (49), volume sharpening (50), local-resolution estimation (51), secondary-structure detection (52), residue-level quality estimation (53) and model building (54,55). EMDB endeavours to continue supporting the development of new ML algorithms through expert curation, and thorough and accurate labelling of entries *via* the EMDB data model.

### Looking forward

As the 3DEM field continues to expand and evolve, the importance of archiving and appropriately describing new types of data will continue to be essential. To this end, EMDB plans to continue to expand the 3DEM data model in collaboration with the community and various wwPDB working groups. Examples of enhancements include provenance description of synthesised macromolecules and labelling of composite maps. Furthermore, as 3DEM experiments are expected to be performed downstream of increasingly heterogeneous preparation methods and/or preceding multimodal imaging experiments, it will be important to be able to capture metadata describing more complex origins of a final 3DEM volume. Planned improvements of metadata describing 3DEM experiments will both support and enhance interoperability of archived data. As an example, metadata is now available in mmCIF format for newly released entries and we plan to expand this to the whole archive, in addition to the existing XML files.

The EMDB Validation Analysis package (36) uses several software tools to generate an extensive set of validation statistics, plots and images, which enable assessment of various volume and map-model features. Comprehensive metrics are important, but the sheer volume of metrics can be overwhelming to some users, particularly those less familiar with 3DEM techniques. Furthermore, calculating an ever-expanding set of metrics for every entry in the exponentially growing archive is a significant computational burden. Future thought will need to be aimed at summarising important results from the Validation Analysis pipeline, whilst limiting the computational and environmental costs of running the software. Summarising results is likely to take a similar form to the ‘sliders’ already presented within wwPDB validation reports for atomic models deposited to the PDB archive (56).

Validation of deposited metadata is essential for ensuring that the archive retains its fidelity and value in the long term. wwPDB has implemented a number of 3DEM-related checks within the OneDep system to improve data reliability. For example, minimum defocus values can no longer be greater than maximum defocus values, and provided pixel sizes of volumes are compared with those reported in the volume header. Additionally, deposition of atomic coordinates that include parts of structures extending beyond the deposited volume's bounding box is now stopped at the file-upload stage. Several multi-file checks are in development, including assessment of whether uploaded half-maps are identical, or whether the primary map and half-maps are offset from one another or have different grid parameters. In rare cases, depositors accidentally select the wrong modality at the start of the deposition process, this results in the instantiation of metadata items designed for a different modality. To mitigate this issue, EMDB is experimenting with deep-learning approaches to predict the experimental method from which a volume was derived. Taken together, these checks will help to ensure that data and metadata archived in EMDB are as accurate as possible and adhere to the FAIR principles.

Finally, developing the EMDB archive to accommodate 3DEM volumes with metadata captured accurately, automatically and robustly validated, with increased description of a dataset's experimental context, will enable the archive to support the field going forward. We expect to support new technologies that generate 3DEM volumes in new ways and to more deeply integrate these data with other structural and non-structural archives. Together, these improvements will support insight into macromolecules in their experimental and biological context, maintaining an archive of high-value to the 3DEM, data science and machine learning communities.

## Data Availability

The data underlying Figure 1 are available in the EMDB* and PDB** archives. The data underlying Figures 2 and 4 are available in the EMDB* archive. The data underlying Figure 3 will be shared on reasonable request to the corresponding author. * accessible at ‘https://ebi.ac.uk/emdb’. ** accessible at ‘https://www.rcsb.org/stats’.

## Notes

Authors details present in appendix.

## Appendix

Current wwPDB Consortium Members with Affiliations

EMDB

Jack Turner^1^, Sanja Abbott^1^, Neli Fonseca^1^, Ryan Pye^1^, Lucas Carrijo^1^, Amudha Kumari Duraisamy^1^, Osman Salih^1^, Zhe Wang^1^, Gerard J. Kleywegt^1^, Kyle L. Morris^1,*^, Ardan Patwardhan^1,*^

RCSB PDB

Stephen K. Burley^2,3,4,5^, Gregg Crichlow^2^, Zukang Feng^2^, Justin W. Flatt^2^, Sutapa Ghosh^2^, Brian P. Hudson^2^, Catherine L. Lawson^2^, Yuhe Liang^2^, Ezra Peisach^2^, Irina Persikova^2^, Monica Sekharan^2^, Chenghua Shao^2^, Jasmine Young^2^

PDBe

Sameer Velankar^6^, David Armstrong^6^, Marcus Bage^6^, Wesley Morellato Bueno^6^, Genevieve Evans^6^, Romana Gaborova^7^, Sudakshina Ganguly^6^, Deepti Gupta^6^, Deborah Harrus^6^, Ahsan Tanweer^6^, Manju Bansal^8^, Vetriselvi Rangannan^8^

PDBj

Genji Kurisu^9,10^, Hasumi Cho^9^, Yasuyo Ikegawa^9^, Yumiko Kengaku^10^, Ju Yaen Kim^10^, Satomi Niwa^10^, Junko Sato^10^, Ayako Takuwa^10^, Jian Yu^10^

BMRB

Jeffrey C. Hoch^11^, Kumaran Baskaran^11^

PDBc

Wenqing Xu^12,13^, Weizhe Zhang^12^, Xiaodan Ma^12^

1. Cellular Structure and 3D Bioimaging, European Molecular Biology Laboratory, European Bioinformatics Institute (EMBL-EBI), Wellcome Genome Campus, Hinxton, Cambridgeshire CB10 1SD, United Kingdom

2. Research Collaboratory for Structural Bioinformatics Protein Data Bank, Institute for Quantitative Biomedicine, Rutgers, The State University of New Jersey, Piscataway, NJ 08854, USA

3. Department of Chemistry and Chemical Biology, Rutgers, The State University of New Jersey, Piscataway, NJ 08854, USA

4. Rutgers Cancer Institute of New Jersey, Rutgers, The State University of New Jersey, New Brunswick, NJ 08901, USA

5. Research Collaboratory for Structural Bioinformatics Protein Data Bank, San Diego Supercomputer Center, University of California San Diego, La Jolla, CA 92093, USA

6. Protein Data Bank in Europe, European Molecular Biology Laboratory, European Bioinformatics Institute (EMBL-EBI), Wellcome Genome Campus, Hinxton, Cambridgeshire CB10 1SD, United Kingdom

7. CEITEC - Central European Institute of Technology, Masaryk University, Kamenice 5, 62500 Brno, Czech Republic

8. Molecular Biophysics Unit, Indian Institute of Science, Bangalore. India

9. Protein Data Bank Japan, Protein Research Foundation, Minoh, Osaka 562–8686, Japan

10. Protein Data Bank Japan, Institute for Protein Research, Osaka University, Suita, Osaka 565–0871, Japan

11. Biological Magnetic Resonance Data Bank, Department of Molecular Biology and Biophysics, UConn Health, 263 Farmington Ave., Farmington, CT 06030,–3305, USA

12. National Facility for Protein Science, Shanghai Advanced Research Institute, Chinese Academy of Sciences, Shanghai 201210, China

13. School of Life Science and Technology, ShanghaiTech University, Shanghai 201210, China
