# Correction to ‘EMDB—the Electron Microscopy Data Bank’

**DOI:** 10.1093/nar/gkad1199

**Published:** 2023-12-07

*Nucleic Acids Research*, 2023, gkad1019, https://doi.org/10.1093/nar/gkad1019

In the originally published version of this manuscript, in the Appendix section, a member of the wwPDB Consortium, Ryan Pye, was inadvertently omitted from the EMDB list.

This has been corrected.

The updated EMDB list now reads as follows:

EMDB

Jack Turner^1^, Sanja Abbott^1^, Neli Fonseca^1^, Ryan Pye^1^, Lucas Carrijo^1^, Amudha Kumari Duraisamy^1^, Osman Salih^1^, Zhe Wang^1^, Gerard J. Kleywegt^1^, Kyle L. Morris^1,*^, Ardan Patwardhan^1,*^.

